# Urine Nerve Growth Factor May Not Be Useful as a Biomarker of Overactive Bladder in Patients with Pelvic Organ Prolapse

**DOI:** 10.3390/jcm11040971

**Published:** 2022-02-13

**Authors:** Katarzyna Jankiewicz, Michał Bogusiewicz, Łukasz Nowakowski, Tomasz Rechberger, Artur Rogowski, Pawel Miotla

**Affiliations:** 1 2nd Department of Gynecology, Medical University of Lublin, 20-954 Lublin, Poland; mbogusiewicz@yahoo.com (M.B.); rechbergt@yahoo.com (T.R.); pmiotla@wp.pl (P.M.); 2 1st Military Clinical Hospital with Polyclinic, 20-049 Lublin, Poland; luknowakow@gmail.com; 3 Department of Minimally Invasive and Endoscopic Gynecology, Military Institute of Medicine, Legionowo Hospital, 05-119 Legionowo, Poland; arogowski@op.pl

**Keywords:** overactive bladder, nerve growth factor, creatinine, biomarkers, detrusor overactivity, pelvic organ prolapse

## Abstract

(1) Background: Overactive bladder (OAB) symptoms are frequently present in women with pelvic organ prolapse (POP). Although urinary nerve growth factor (NGF) is a promising biomarker of OAB, little is known about its role in patients with OAB secondary to POP. The aim of the study was to evaluate urinary NGF levels in patients with POP involving the anterior vaginal wall and check if it may serve as a predicting factor for postoperative resolution of OAB symptoms. (2) Methods: Eighty-three Caucasian women included in the study were divided into three groups: pure OAB, one associated with POP (POP&OAB) and a control group composed of healthy volunteers. The urine NGF and creatinine were assessed with ELISA tests to calculate the NGF/creatinine ratio. (3) Results: The NGF/creatinine ratio was significantly higher in patients with pure OAB in comparison with other groups; however, it did not differ between the control group and the POP&OAB group. There was no correlation between NGF/creatinine ratio and age, menopausal status, BMI, parity or urodynamic findings. The NGF/creatinine ratio was not a prognostic factor for OAB symptoms’ resolution after surgical treatment of POP. (4) Conclusions: Urinary NGF excretion is not increased in women with OAB secondary to POP; thus, it may not serve as an OAB biomarker in these patients.

## 1. Introduction

Overactive bladder (OAB) is a condition characterized by symptoms of urgency and frequency with or without urge incontinence in the absence of other proven pathologies. Diagnosis is based on medical history and the analysis of the micturition diary [1]. Urodynamic study (UDS), although not required in the diagnostic flow of an OAB patient, may provide more objective data by detecting detrusor overactivity (DO) as well as assessing maximum flow rate (Qmax), maximum cystometric capacity (MCC), voided volume, and detrusor pressure at maximal flow rate (PdetQmax). Furthermore, it excludes overflow incontinence and other pathologies that mimic OAB and confirms urodynamic stress urinary incontinence (SUI) [2]. Urodynamic diagnosis of OAB is based on the finding of bladder contraction (DO); however incidence of DO among OAB patients varies in the range of 10–38% [3].

Regarding low sensitivity of UDS, other objective tools, such as assessment of biomarkers, have been considered. Among many options investigated, the nerve growth factor (NGF) seems to meet the criteria of a potent OAB biomarker [4,5,6]. Since blood NGF levels can be raised in cardiovascular diseases, bladder cancer, and bladder nephrolithiasis, as well as some neurological diseases or diabetes, it appears that the most specific and sensitive method is assessment of urine NGF. Based on the finding that urinary NGF concentration may be affected by urine-specific gravity, it has been proposed that measurement of the NGF/creatinine ratio is the most accurate method [5]. Many studies indicate that urinary NGF/creatinine levels are significantly higher in symptomatic OAB patients compared to healthy individuals [4,7]. Furthermore, NGF urine levels decrease after effective pharmacological treatment of OAB [8] and correlate to OAB type—patients with OAB wet have significantly higher urinary NGF levels than patients with OAB dry [9]. 

OAB symptoms are often seen in patients with pelvic organ prolapse (POP). Prevalence depends on the compartment affected and the stage of prolapse, but overall, ranges from 16% to 88% [10]. There are several theories about OAB pathophysiology in women with POP. The most accepted is the one attributing the development of OAB symptoms in patients with prolapse to bladder outlet obstruction (BOO). Interestingly, there are several animal and human studies indicating that BOO stimulates NGF release by bladder wall cells and increases its urine levels [10]. However, due to scarcity of data regarding POP patients, it is unclear if NGF may serve as a useful biomarker in this group of women. 

The present study was designed to measure the urinary NGF/creatinine ratio in women with idiopathic OAB, OAB accompanying POP and healthy controls. We aimed to check if the urinary NGF/creatinine ratio may be used as a potential biomarker in women with OAB symptoms secondary to POP. Moreover, it was of our interest to verify the hypothesis that the baseline value of the NGF/creatinine ratio may serve as a prognostic factor for OAB symptoms’ resolution after surgical treatment of prolapse. 

## 2. Materials and Methods

**Figure 1:**
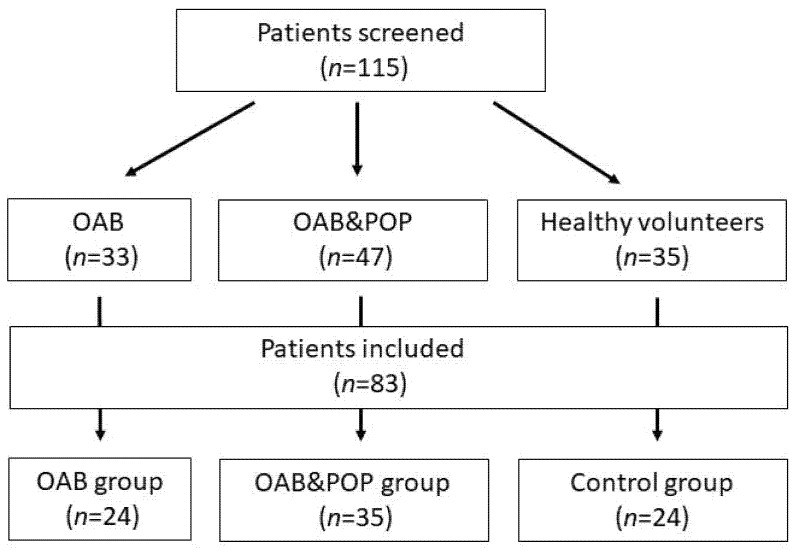
Flowchart shows number of patients screened and included in the study. OAB—pure OAB, POP&OAB—pelvic organ prolapse with urgency.

A total number of 115 Caucasian women (80 with OAB and 35 controls) were screened (Figure 1). Eighty-three fulfilled the criteria of our study. OAB diagnosis (including idiopathic OAB or OAB with POP) was based on the presence of urgency with or without urge incontinence, frequency and nocturia and a three-day voiding diary (≥8 micturition per 24 h) [11,12,13,14]. The patients with OAB were divided into two groups: pure OAB (OAB group, *n* = 24) and pelvic organ prolapse with urgency (POP&OAB group, *n* = 35). Twenty-four healthy volunteers without POP or lower urinary tract symptoms (LUTS) composed the control group. Each type of LUTS (OAB, POP) was diagnosed following International Continence Society (ICS) guidelines [11]. The urodynamic studies were conducted following Good Urodynamic Practices guidelines. An ICS standard urodynamic test (ICS-SUT) consisting of uroflowmetry, PVR, transurethral cystometry and pressure-flow study was performed in each OAB [15]. We excluded patients with a medical history of urogynecological surgery, including anti-incontinence procedures, or a neurogenic bladder, diabetes, asthma, neurological diseases, pharmacological treatment of OAB at enrolment, mixed or stress urinary incontinence, overflow incontinence, pregnancy, and gynecologic conditions affecting urinary incontinence (UI) despite POP in the POP&OAB group. The extent of prolapse was evaluated using Pelvic Organ Prolapse Quantification [16]. The POP&OAB group comprised patients with symptomatic anterior vaginal wall prolapse at stage II or III and posterior wall prolapse not larger than stage II. 

Urinary NGF and creatinine levels were measured by enzyme-linked immunosorbent assay (ELISA) according to the manufacturers’ instructions. The urine NGF and creatinine were assessed in the same sample collected during the midstream second-morning voiding. Voided urine was centrifuged at 3000× *g* for 10 min at 4 °C. Then, the supernatant was separated in aliquots of 1.5 mL and stored at −80 °C. 

The ELISA tests were used; for NGF-Human, the NGF ELISA kit was used (My Bio Source, San Diego, CA, USA, Catalog No.: MBS355238); for creatinine, the ELISA kit was used (RnD Systems, Minneapolis, MN, USA, Catalog No.: KGE005). The urinary NGF/creatinine levels were compared among the control, pure OAB, and OAB coexisting with POP groups. We also assessed NGF levels according to age, menopausal status, parity, BMI, UDS (DO, MCC) and in the POP&OAB group effect of surgery on OAB symptoms three months after.

Statistical analysis was performed with Statistica StatSoft 13.0 (StatSoft, Tulsa, OK, USA). A Shapiro–Wilk test and a Lilliefors test were used to verify normality within the groups. In case of normal distribution (age of the patients), a one-tailed ANOVA test was applied. For nonparametric statistics (NGF/creatinine ratio), the Kruskal–Wallis test and Spearman’s correlation coefficient were used to verify statistical hypotheses as appropriate. Analysis of categorical data was performed with the chi^2^ test. Continuous variables are expressed as the mean ± standard deviation or the median, whereas categorical data are expressed as frequency and percentage. A value of *p* < 0.05 was considered statistically significant.

## 3. Results

Table 1 shows demographic and clinical data. The patients from the OAB group were younger in comparison to the other two groups, which reflects the fact that idiopathic OAB is typically diagnosed earlier than that associated with POP. We matched the age of control patients with the POP&OAB group. 

**Figure 2:**
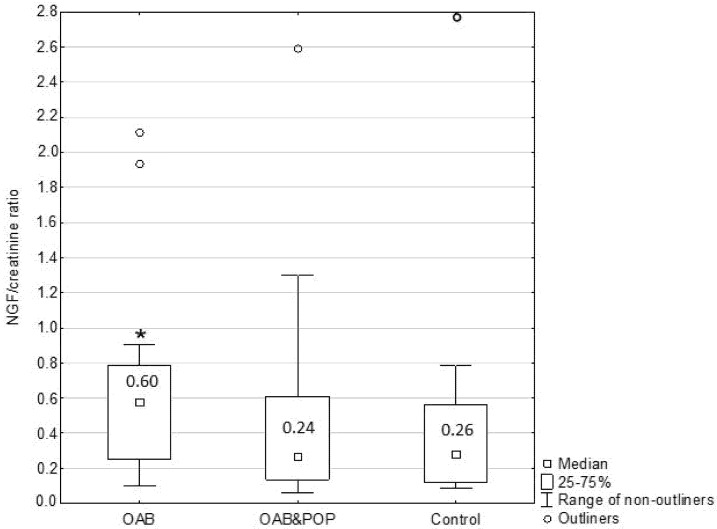
Urine NGF/creatinine ratio in the study groups. The mean urine NGF/creatinine ratio was higher in the OAB group than in the POP&OAB and control groups (*p* < 0.05); however, it did not differ significantly between POP&OAB and control patients (*).

The mean NGF/creatinine ratio was higher in the OAB group than in the POP&OAB and the control groups; however it did not differ significantly between POP&OAB and control patients (Figure 2).

To check the role of age as a confounding factor potentially affecting study results, we correlated the age of all study participants with the NGF/creatinine ratio. We did not find a statistically significant correlation between these variables, and thus it is unlikely that the age of participants affected the results of our study. Similarly, the analysis of the NGF/creatinine ratio in relation to BMI and parity revealed no correlations. It also was not related to menopausal status. Thus, it appears that these variables did not influence the study results. Furthermore, the NGF/creatinine ratio was not correlated with urodynamic parameters (DO and MCC) (Table 2). The median value of the NGF/creatinine ratio was not statistically higher in patients with DO (*n* = 11) in comparison with patients without DO (*n* = 48) (0.46 vs. 0.30).

Three months after POP surgery, patients with OAB were reassessed. Seventeen (48.6%) patients reported resolution or significant improvement of symptoms; however, the baseline value of the NGF/creatinine ratio did not appear to be a predictive factor (0.38 ± 0.30 vs. 0.45 ± 0.59).

## 4. Discussion

In the urinary tract NGF is produced by the urothelium and smooth muscle of the bladder, and regulates the subpopulation of peptidergic bladder afferent sensory fibers. NGF production increases due to inflammation, spinal cord injury, denervation and BOO [17]. Neurotrophins, including NGF, in the bladder work through two classes of receptors: TrkA receptors (the high-affinity tropomyosin-related kinase A) and p75NTR (the low-affinity p75 neurotrophin receptor) [18]. NGF is one of the substances that increases sensory C-fiber activity. In rats, intravesical administration of NGF caused bladder contractions [19]. NGF levels modulation by blocking TrkA or NGF sequestration restores proper bladder function [20]. NGF also regulates TRPV1 receptors, which are known as important for sensory activity. Interestingly, in BOO, patients’ bladders exhibit lower density of nerves compared to healthy ones [17]. 

According to de Boer et al., prevalence of OAB in women with POP depends on the compartment and stage but ranges on total from 16% to 88% [10]. Furthermore, over 90% of women with OAB have anterior vaginal wall descent [10]. Although OAB symptoms are frequently seen in patients with POP, the pathogenesis of OAB is not completely clear. One hypothesis attributes the occurrence of OAB to bladder outlet obstruction. In animal models, an obstruction causes hypertrophy of the afferent neurons and results in an increase of NGF in the bladder wall [17]. Another theory links OAB symptoms to pelvic ligaments laxity. If the ligaments are lax the vaginal membrane does not support the stretch receptors. In the bladder base the hydrostatic pressure of the urine stimulates stretch receptors and prematurely activates micturition reflex. It is also considered that OAB associated with POP can be caused by traction of the urethra through cystocele and urine entering the urethra [21].

In our study, urinary NGF/creatinine levels were significantly higher in women with OAB when compared to healthy individuals as well as when compared to the POP&OAB group. There was no statistically significant difference in the mean NGF/creatinine ratio between patients with prolapse and the control group. These findings support the theory that urinary NGF may be an OAB biomarker, but however may not be reliable in the case of anterior vaginal wall descent. Yildiz et al. showed that NGF blood levels were highly elevated in patients with significant prolapse (stages 3 and 4) and OAB [22]. Since many studies link increased production of NGF to BOO, this observation suggests that BOO is the main pathophysiological mechanism responsible for development of OAB in women with prolapse. On the contrary, our findings of elevated NGF/creatinine levels only in patients with idiopathic OAB suggest a different pathological background in the case of OAB associated with POP. It is possible that BBO does not play a pivotal role in the development of OAB in cases of less pronounced prolapse. However, more detailed investigation is required to verify this hypothesis. We did not analyze the relationship between severity of BOO and the NGF/creatinine ratio, which is apparently a limitation of this study. 

Other limitations are the single-institution setting and relatively small sample size. Furthermore, confirmation of ELISA results with other protein analysis methods such as a multiplex assay would increase the robustness of the study. 

We did not observe any relationship between NGF/creatinine levels and aging, menopause or higher BMI, neither in the control group nor in OAB patients. Similarly, Pennycuff et al. found no difference in urinary levels of NGF between postmenopausal OAB women and age-matched controls, though they observed a correlation between higher urinary NGF/creatinine levels and increasing age and BMI [23]. Association of NGF levels with increasing age of women was also seen in other studies [24].

We also checked if the baseline NGF/creatinine ratio may be a predictive factor for successful OAB symptoms’ resolution after surgical treatment of prolapse. However, the results of the study failed to support this assumption.

The diagnosis of OAB is usually based on subjective symptoms reported by the patient. Many studies identified urinary levels of NGF as a potential biomarker and a non-invasive, cost-effective method to confirm OAB diagnosis as well as to assess therapeutic outcomes. Urinary NGF may also serve as a prognostic indicator of treatment. However, since NGF levels may be elevated in numerous conditions, further investigations need to be conducted to establish the value of this biomarker in clinical practice. NGF mediates bidirectional communication between the muscle or urothelium and nerves contributing to the development of OAB. Further studies on the role of NGF should provide valuable insights into the pathogenesis of this condition [25,26].

## 5. Conclusions

Our study showed that the urinary NGF/creatinine ratio may be a potent biomarker for idiopathic OAB. However, since urinary NGF excretion is not increased in women with OAB secondary to POP, the NGF/creatinine ratio cannot serve as an OAB biomarker in these patients. Furthermore, our findings suggest that the pathogenesis of OAB in some patients with POP may differ from those with underlying pure OAB.

## Figures and Tables

**Table 1:** Demographic and clinical characteristics of the study groups. The patients from the OAB group were younger (*p* < 0.05) in comparison to other two groups (*).

	OAB (*n* = 24)	POP&OAB (*n* = 35)	Control (*n* = 24)
Age (years, mean ± SD)	55.9 ± 12.8 *	61.3 ± 9.0	61.3 ± 11.7
Menopause (*n*, %)	14 (58.3)	29 (82.9)	16 (66.7)
BMI (kg/m^2^, mean ± SD)	26.8 ± 4.2	29.2 ± 4.3	27.6 ± 4.4
Parity (*n*, mean ± SD)	2.2 ± 0.8	2.3 ± 1.0	2.1 ± 1.2
Detrusor overactivity (*n*, %)	3 (12.5)	8 (22.9)	0

**Table 2:** Correlations between NGF/creatinine ratio and age, parity, BMI and MCC (Spearman’s rank-order correlation).

	Overall R (*p*)	OAB R (*p*)	POP&OAB R (*p*)	CONTR R (*p*)
NGF/creatinine and Age	−0.07 (0.54)	−0.07 (0.6)	0.04 (0.82)	0.08 (0.7)
NGF/creatinine and Parity	−0.04 (0.71)	−0.04 (0.86)	0.00 (0.99)	−0.15 (0.5)
NGF/creatinine and MCC	−0.17 (0.24)	−0.17 (0.21)	0.07 (0.68)	NA
NGF/creatinine and BMI	−0.03 (0.81)	−0.03 (0.56)	0.01 (0.96)	−0.04 (0.84)

## Data Availability

Data supporting reported results can be found in our local database.
